# Rhode Island gastroenterology video capsule endoscopy data set

**DOI:** 10.1038/s41597-022-01726-3

**Published:** 2022-10-06

**Authors:** Amber Charoen, Averill Guo, Panisara Fangsaard, Supakorn Taweechainaruemitr, Nuwee Wiwatwattana, Theekapun Charoenpong, Harlan G. Rich

**Affiliations:** 1 The Warren Alpert Medical School of Brown University, Division of Gastroenterology, Providence, Rhode Island 02903 United States of America; 2 Chulabhorn Hospital, Chulabhorn Royal Academy, Bangkok, 10210 Thailand; 3 Srinakharinwirot University, Department of Computer Science, Faculty of Science, Bangkok, 10110 Thailand; 4 Srinakharinwirot University, Department of Biomedical Engineering, Faculty of Engineering, Nakhonnayok, 26120 Thailand

**Keywords:** Oesophagus, Stomach, Small intestine

## Abstract

Complete endoscopic evaluation of the small bowel is challenging due to its length and anatomy. Although several advances have been made to achieve diagnostic and therapeutic goals, including double-balloon enteroscopy, single-balloon enteroscopy, and spiral enteroscopy, video capsule endoscopy (VCE) remains the least invasive tool for complete visualization of the small bowel and is the preferred method for initial diagnostic evaluation. At present, interpretation of VCE data requires manual annotation of landmarks and abnormalities in recorded videos, which can be time consuming. Computer-assisted diagnostic systems using artificial intelligence may help to optimize VCE reading efficiency by reducing the need for manual annotation. Here we present a large VCE data set compiled from studies performed at two United States hospitals in Providence, Rhode Island, including 424 VCE studies and 5,247,588 total labeled images. In conjunction with existing published data sets, these files may aid in the development of algorithms to further improve VCE.

## Abstract (summary)

**Table:** 

Measurement(s)	organ
Technology Type(s)	Videocapsule Endoscopy
Sample Characteristic - Organism	Homo sapiens
Sample Characteristic - Location	State of Rhode Island

## Background & Summary

The small intestine is about 6 m in length and is the longest organ in the gastrointestinal (GI) tract. It begins at the gastric pylorus and ends at the ileocecal valve, and is comprised of three segments known as the duodenum, jejunum, and ileum. The duodenum is about 25 cm in length and is divided into four sections known as the bulb, second or descending portion, third or transverse or horizontal portion, and fourth or ascending portion. The jejunum and ileum together measure about 5 m. Functionally, the small intestine is responsible for digestion and nutrient absorption. This is accomplished through a unique mucosal lining composed of finger-like projections, called villi, which augments the small intestine surface area by 600- to 1000-fold, totaling 250 to 400 m^2^ ^1^. Disorders affecting the small intestine can be quite debilitating, often resulting in significant nutritional deficiencies and diarrheal illness, and can include Celiac disease, Crohn’s disease, malignancies, and other diseases of vascular and infectious etiologies. Gastrointestinal bleeding from the small intestine is infrequent, accounting for an estimated 3–5% of GI bleeds, and is often challenging to diagnose and treat^2^. Video capsule endoscopy (VCE) is used in the assessment of obscure GI bleeding and as a complementary diagnostic tool in other small bowel disorders when other endoscopic and imaging modalities are unrevealing.

VCE is usually performed via the use of a swallowed or endoscopically placed disposable plastic capsule containing a battery, camera, transmitter, and light source, which wirelessly transmits images via a sensor array belt worn by the patient to a data recorder; one system stores the images with the camera and downloads them later for review. Several VCE systems have been developed by manufacturers including Olympus America, Given Imaging, Intromedic Company, Ankon Technologies, Chongqing Science, and CapsoVision^3–5^. Technological improvements have allowed for wider fields of view (140°–360°), improved battery life, increased numbers of enclosed cameras, and variable frame rates (2–35 frames per second) that can be adjusted based on the speed of transit through the GI tract, capturing fewer images with slow transit and more images with faster transit^5^. Each system uses proprietary software to process images for review by the physician^5^. While handheld viewers allow for real-time review of images with some systems, VCE studies are most often reviewed after the capsule has passed completely through the small intestine. Ultimately, 8–12 hours of video and over 50,000–60,000 images may be generated in an individual study^5^. Image review is aided by software that allows for grouped viewing of 2–4 images at one time. Red pixel detection can also help to identify possible areas of bleeding. VCE image assessment remains time consuming. Review for the identification of anatomical landmarks and abnormalities requires up to 30–90 minutes per study. Fatigue related to the sustained and prolonged degree of concentration necessary to review such a large number of images may lead to errors, missed lesions, as well as inter- and intra-observer variation.

Computer-assisted diagnostic systems using artificial intelligence may help to address these challenges through automated data analysis, reducing VCE study assessment time, limiting required manual annotations, and improving both lesion detection and capsule localization^3^. Annotated VCE data sets may help to achieve this by aiding in the development of machine learning algorithms. There are currently few such VCE data sets, and most prioritize lesion detection^3,6^. Here we present the Rhode Island Gastroenterology VCE data set, a publicly available annotated data set, which contains 424 videos with 5,247,588 total labeled images denoting 4 anatomic classifications. To our knowledge, this is the largest such public data set currently available. While not every video in this data set was completely normal, annotations of abnormalities are not included here. Our focus was instead on landmark identification, a key initial step in the assessment of VCE studies which sets the foundation for correctly identifying and locating abnormalities^7,8^. Identifying abnormalities on VCE without adequate localization within the GI tract limits the ability to direct subsequent endoscopic interventions. This is particularly true in the small bowel where precise localization remains challenging and even minor improvements may be beneficial. Furthermore, machine learning guided identification of landmarks may help to limit variability among physicians with varying levels of expertise, thus leading to improved standardization^9^. Finally, identifying landmarks of segments within the GI tract at present requires a significant amount of manual annotation. Reducing the time required for this will improve the efficiency of VCE study interpretation^10^. Our hope is that this current data set will be most useful in improving landmark identification by artificial intelligence systems. Additional future endeavors to annotate abnormalities in this data set may help to improve artificial intelligence assisted lesion detection.

## Methods

### Data collection

The Rhode Island GI VCE data set was obtained from the VCE procedures performed at Rhode Island Hospital (RIH) and The Miriam Hospital (TMH) from January 2016 to December 2020. The VCE device used was the PillCamTM SB3 capsule, which was used together with its belt and recorder. Following complete image collection, images were downloaded for review and de-identified. Several patients underwent more than one VCE study, however, due to the de-identification process, these particular studies cannot be identified and were each treated as unique studies. This project was reviewed by the institutional review board of RIH and TMH (RIH IRB 1: IRB00000396, RIH IRB 2: IRB00004624, and TMH IRB: IRB00000482). As the project was granted a waiver of informed consent, these data were retrospectively obtained without the patients’ written consent. There is no protected health information contained in the data set and there are no restrictions on the use or disclosure of de-identified health information^11^. As such, these data may be shared under the Health Insurance Portability and Accountability Act Privacy Rule (HIPAA Privacy Rule).

### Data de-identification

The RAPIDTM Reader or PillcamTM software is developed and supplied by the device manufacturer (Medtronic), and is used to open the proprietary video file downloaded from the PillCamTM system. To remove personal patient information from the video files, capsule studies were exported using the de-identify function in the RAPID reading software.

### Data conversion

Following the de-identification process, the exported files remain in the proprietary video format. As a result, they must be converted into images (.png) using FFmpeg software (available from http://ffmpeg.org/). The original resolution is 320 × 320 pixels.

### Data labeling

VCE images were classified into four classes by anatomical organ, including esophagus, stomach, small bowel, and colon. We identified the first image of each organ and the last image of each organ (the image before the first image of the next organ, except in the case of the colon). All images from the first image to the last image of each organ were labeled accordingly. The images that could not be classified into one of the four above described anatomical organs were removed. The majority of the removed images were before the first image of esophagus. To ensure the quality of the data set, the data were reviewed by two physicians specializing in gastroenterology. The first physician labeled the first images of each anatomical organ during the standard process of VCE interpretation. The second physician reviewed and validated the initial results. If there were any differences, a consensus between the two physicians was reached.

### Inclusion and exclusion criteria

We only included VCE studies in which patients swallowed the capsule themselves. Endoscopy-deployed VCE studies were excluding from this data set. Furthermore, we only included VCE studies in which the images could be sequentially labeled into four anatomical organs.

## Data Records

**Fig. 1:**
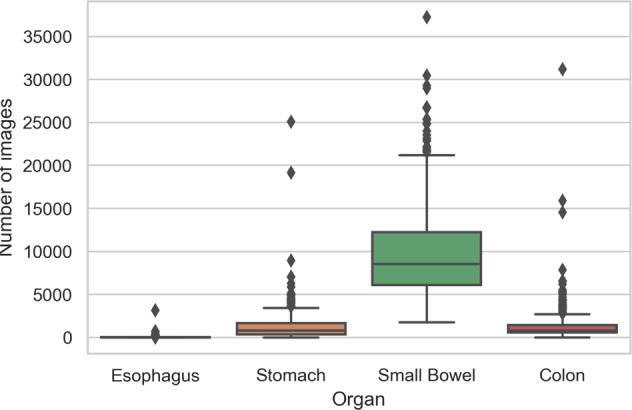
The box plot of number of images per anatomical organ for a study.

**Fig. 2:**
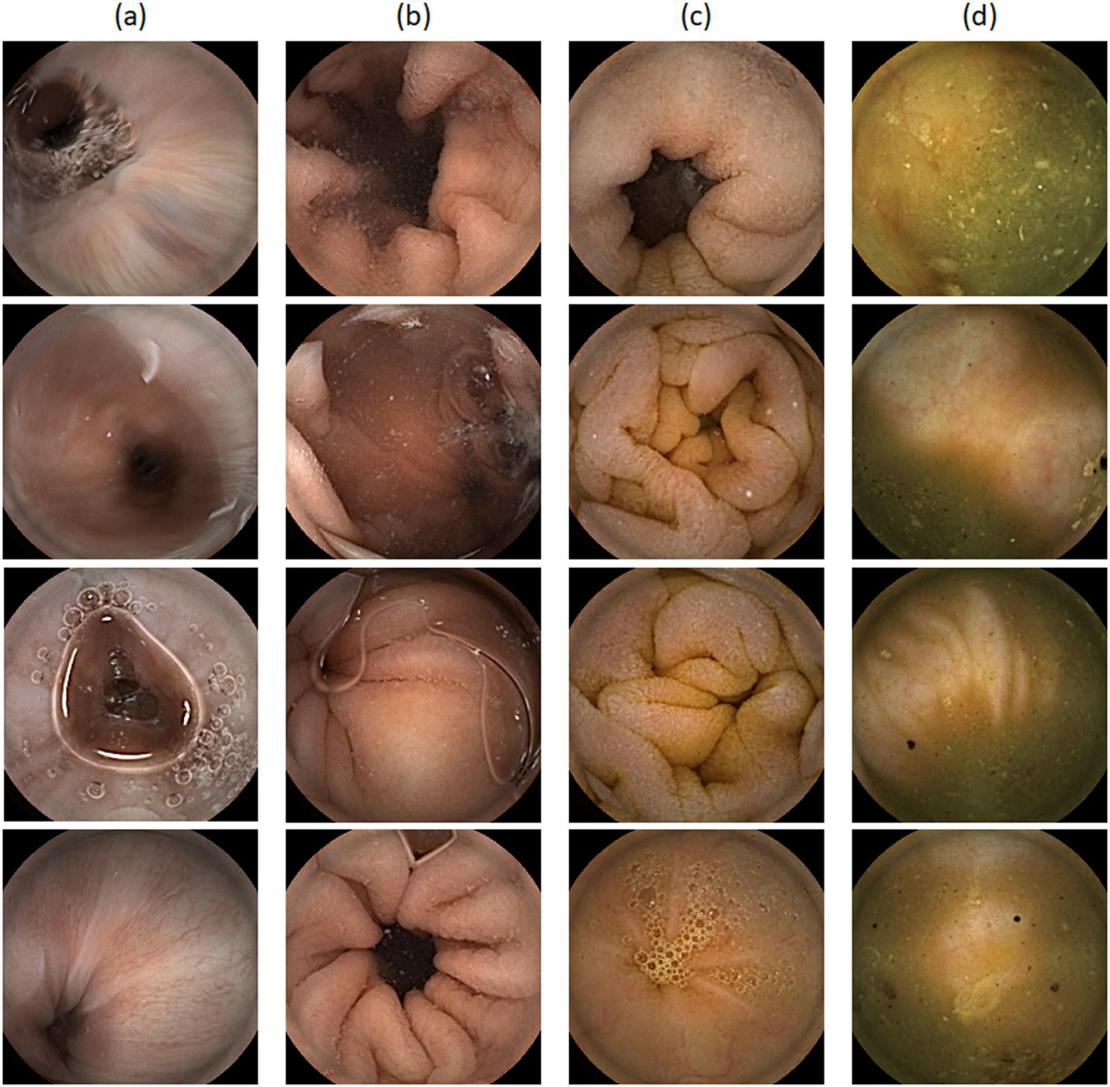
The sample images of four anatomical organs: (**a**) esophagus, (**b**) stomach, (**c**) small bowel, and (**d**) colon.

In the Rhode Island GI VCE data set, there are 424 VCE studies consisting of 5,247,588 images in total. Each study is saved in the form of a zip file containing four sub-folders for each anatomical organ (1 esophagus, 2 stomach, 3 small bowel, and 4 colon); each sub-folder represents a labeled anatomical organ in the gastrointestinal tract. The average number and total number of images per anatomical organ are presented in Fig. 1 and Table 1. The representative images for each anatomical organ shown in Fig. 2 were taken from the first sample (s001). These data are available at Figshare^12^.

**Table 1:** The descriptive statistics of the data set (number of images per anatomical organ).

	Esophagus	Stomach	Small bowel	Colon
Minimum	1	1	1,737	1
1st quartile	6	372	6,091	558
Median	12	796	8,537	825
3rd quartile	21	1,657	12,249	1,445
Maximum	3,152	25,081	37,240	31,184
Mean	32	1,314	9,698	1,332
Total	13,715	557,049	4,111,865	564,959

## Technical Validation

To provide a baseline performance for the classification model, we used the Inception ResNet V2 architecture to create a predictive model based on a down-sampling training set^13^. Inception Resnet V2 is among the most popular models for image classification that can provide very good performance and convergence speed, which are derived from the strengths of the inception model and residual networks. Based on the Pareto principle (80/20 rule), the data set of 424 samples was randomly separated into (1) training/validation set of 339 samples (80%) and 4,193,038 images (79.9%), and (2) testing set of 85 samples (20%) and 1,054,550 images (20.1%). In the training/validation set, the smallest class is esophagus with 9,061 images (0.2%). Since the minority class is less than 1% of the total number of images, the degree of class imbalance is considered extreme. As a result, we performed down-sampling on other classes to reduce class imbalance issues such that the number of images of the majority classes (stomach, small bowel, and colon) was reduced to 11,508, 32,252, and 11,707 images, respectively. To reduce the degree of imbalance, the down-sampling was done by randomly selecting images (1 out of 400 for small bowel and 1 out of 100 for the two classes) per class in each sample. It is worth noting that the down-sampling provide an additional benefit in reducing the training data size and computational resources for training the deep learning model. Then, we randomly split the training/validation set into a training set (75%) and a validation set (25%); the splitting proportion was set to optimize the training time within a reasonable time period. The number of images per data set is summarized in Table 2. The Inception ResNet V2 model was used to train the model; the trained model was then used to predict the anatomical organs on the full testing set.

**Table 2:** The number of images per data set.

Data set	Esophagus	Stomach	Small bowel	Colon	Total images
Training/validation	9,061	466,562	3,242,639	474,776	4,193,038
- Down-sampling	9,061	11,508	32,252	11,707	64,528
- Training	6,795	8,631	24,189	8,780	48,395
- Validation	2,266	2,877	8,063	2,927	16,133
Testing	4,654	90,487	869,226	90,783	1,054,550

**Fig. 3:**
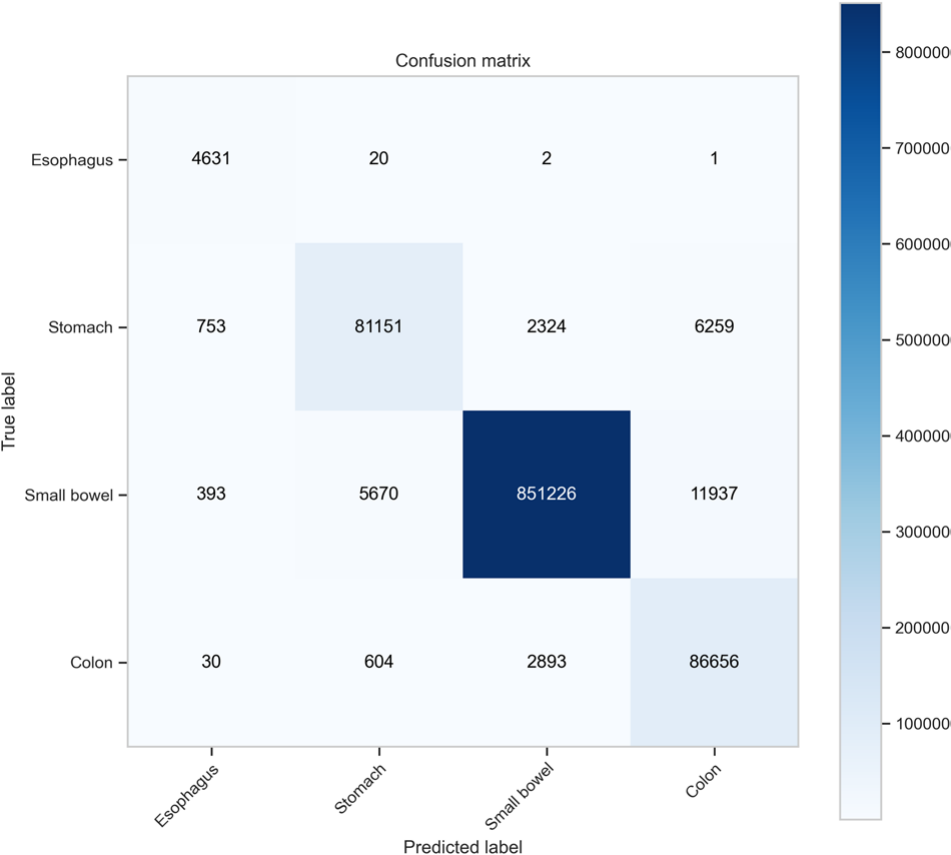
The unnormalized confusion matrix.

The testing performance is presented in Table 3. The overall accuracy is 97.1%, however, the classification performance is highly class dependent. The small bowel, which is often the primary organ under investigation in VCE studies, demonstrates the best performance (precision = 0.9939, recall = 0.9793, and F1 score = 0.9865). Mis-classification (for example, predicting stomach from an image of the colon) may lead to complex problems in practice when attempting to identify the first image of each anatomical organ. The confusion matrix (Fig. 3) shows that the mis-classification rate is relatively low.

**Table 3:** The testing performance of the trained model.

	Precision	Recall	F1 score	Support (images)
Esophagus	0.7975	0.9951	0.8854	4,654
Stomach	0.9280	0.8968	0.9122	90,487
Small bowel	0.9939	0.9793	0.9865	869,226
Colon	0.8265	0.9609	0.8886	90,183
Accuracy			0.9707	1,054,550
Macro average	0.8865	0.9580	0.9182	1,054,550
Weighted average	0.9731	0.9707	0.9713	1,054,550

## Usage Notes

The data set is being shared under the Creative Commons Attribution 4.0 International Public license, which allows users to use, share, adapt, distribute, and reproduce the data set with the appropriate credit given to the original authors and the data source. More details regarding the license can be found in https://creativecommons.org.

The image names in each of the VCE studies are in numerical order. We did not exclude any images from the esophagus to the colon. As a results, some images contain abnormalities such as bleeding, erosions, polypoid lesions, and other GI lesions.

## Data Availability

The code and information needed for training and testing the deep learning model presented in the technical validation section are published on GitHub, which can be accessed via https://github.com/acharoen/Rhode-Island-GI-VCE-Technical-Validation. The pre-trained model used to generate the predictions (InceptionResNetV2.h5) is stored in the same data repository^12^.
